# MRI-based tumor signatures as prognostic biomarkers in oral tongue squamous cell carcinoma

**DOI:** 10.3389/fonc.2026.1809223

**Published:** 2026-04-14

**Authors:** Nivedita Chakrabarty, Nithin Krishnan, Abhishek Mahajan, Tapish Dadlani, Minit Shah, Anuja Deshmukh, Arjun Singh, Sarbani Ghosh Laskar, Gouri Pantvaidya, Ankush Shetake, Swapnil Ulhas Rane, Munita Bal, Deepa Nair, Sudhir V. Nair, Nandini Menon, Asawari Patil, Vanita Noronha, Kumar Prabhash, Pankaj Chaturvedi

**Affiliations:** 1 Department of Radiodiagnosis, Tata Memorial Centre, Advanced Centre for Treatment, Research and Education in Cancer (ACTREC), Homi Bhabha National Institute (HBNI), Mumbai, Maharashtra, India; 2 Department of Radiodiagnosis, Tata Memorial Centre, Homi Bhabha National Institute (HBNI), Mumbai, Maharashtra, India; 3 Department of Imaging, The Clatterbridge Cancer Centre NHS Foundation Trust, Liverpool, United Kingdom; 4 Faculty of Health and Life Sciences, University of Liverpool, Liverpool, United Kingdom; 5 Department of Medical Oncology, Tata Memorial Centre, Homi Bhabha National Institute (HBNI), Mumbai, Maharashtra, India; 6 Department of Head and Neck Surgical Oncology, Tata Memorial Centre, Homi Bhabha National Institute (HBNI), Mumbai, Maharashtra, India; 7 Department of Head and Neck Surgical Oncology, Tata Memorial Centre, Advanced Centre for Treatment, Research and Education in Cancer (ACTREC), Homi Bhabha National Institute (HBNI), Mumbai, Maharashtra, India; 8 Department of Radiation Oncology, Tata Memorial Centre, Homi Bhabha National Institute (HBNI), Mumbai, Maharashtra, India; 9 Department of Clinical Research Secretariat, Tata Memorial Centre, Homi Bhabha National Institute (HBNI), Mumbai, Maharashtra, India; 10 Department of Pathology, Tata Memorial Centre, Homi Bhabha National Institute (HBNI), Mumbai, Maharashtra, India

**Keywords:** imaging biomarker, oral tongue carcinoma, peritumoral edema, peritumoral hyperenhancement, prognostic MRI-based biomarkers, tumor ulceration

## Abstract

**Introduction:**

This study evaluated the prognostic significance of MRI features—peritumoral hyperenhancement, peritumoral hyperenhancement width, peritumoral edema, tumor ulceration, and tumor <5 mm from the hyoid bone—in patients with oral tongue squamous cell carcinoma. Outcomes assessed included overall survival (OS), recurrence-free survival (RFS) [local (LRFS), locoregional (LRRFS), and distant (DRFS)], and disease-free survival (DFS). The secondary objectives included associations with perineural invasion (PNI) and extranodal extension (ENE) on histopathology.

**Materials and methods:**

A retrospective cross-sectional study was conducted at a tertiary cancer center, including 221 treatment-naive oral tongue squamous cell carcinoma patients who underwent surgery between January 2021 and December 2022. Pre-treatment MRIs were reviewed by two blinded radiologists. Patients were followed up for 2 years. Univariate and multivariate analyses were performed. Categorical associations were evaluated using Fisher’s exact or chi-squared tests (*p* < 0.05).

**Results:**

The cohort comprised 221 patients (179 men and 42 women), with a mean age of 47.1 ± 10.88 years. Tumor <5 mm from the hyoid was associated with worse OS [hazard ratio (HR): 3.05; *p* = 0.034], DRFS (HR: 4.66; *p* = 0.041), and DFS (HR: 3.68; *p* = 0.007), peritumoral edema predicted worse DRFS (HR: 4.77; *p* = 0.036), and pathological T categories predicted inferior OS and DFS in multivariate analyses. Peritumoral hyperenhancement and tumor ulceration predicted inferior OS and DFS, respectively, in univariate analyses. These variables were linked to ENE, while peritumoral hyperenhancement width >2.65 mm was associated with PNI.

**Conclusion:**

Peritumoral edema, tumor ulceration, peritumoral hyperenhancement (and width >2.65 mm), and tumor <5 mm from the hyoid bone are adverse MRI biomarkers in oral tongue squamous cell carcinoma, which can refine risk stratification and inform future T4 staging, warranting prospective validation in larger cohorts.

## Introduction

Oral cavity cancer is the 16th most common cancer globally (1). The ninth version of the Union for International Cancer Control (UICC) Tumor Node Metastasis (TNM) staging for head and neck cancers did not alter the oral cavity cancer staging from the eighth edition, except for clarifying that superficial invasion of adjacent skin—such as the dry vermilion and vermilion border of the lip—from the mucosal lip is not enough to classify a tumor as T4a (2, 3). Surgery is the mainstay of treatment for oral tongue cancer, which has an unfavorable prognosis even in the early stages due to high locoregional recurrence (4–6). Tumor recurrence occurs in 10%–25% of early-stage cases, rising to 40%–60% in advanced stages, and is associated with poor overall survival (OS) (7, 8). Depth of invasion (DOI) and extranodal extension (ENE) are well-established prognostic indicators and have therefore been incorporated in the eighth edition of the American Joint Committee on Cancer (AJCC) TNM staging for oral cavity cancers (2, 9). Studies have also shown that the presence of perineural invasion (PNI) on post-operative histopathology is a poor prognostic factor (10, 11).

It is essential to identify pre-operative imaging features that can serve as biomarkers of poor prognosis for oral tongue squamous cell carcinoma (OTSCC). This would allow for the planning of intensive adjuvant therapy and personalized, more frequent follow-up schedules based on pre-operative magnetic resonance imaging (MRI).

The primary objectives of our study were to evaluate the role of peritumoral hyperenhancement, maximum peritumoral hyperenhancement width, peritumoral edema, tumor ulceration, and extension of tumor up to <5 mm from the hyoid bone on pre-operative treatment-naive MRI images of OTSCC in predicting OS, local recurrence-free survival (LRFS), locoregional recurrence-free survival (LRRFS), distant recurrence-free survival (DRFS), and disease-free survival (DFS).

The secondary objectives were to evaluate the association of the above-mentioned imaging parameters with PNI and ENE on post-operative histopathology for OTSCC.

## Materials and methods

### Study design and setting

A retrospective observational cross-sectional study was conducted in a tertiary cancer hospital. Treatment naive MRI of upfront operated patients with OTSCC between 01/01/2021 and 31/12/2022 were evaluated after obtaining Institutional Ethical Committee clearance. A total of 221 patients who fulfilled the inclusion and exclusion criteria were enrolled via hospital electronic medical records (EMRs). Post-operative pathological and follow-up data of 2 years were obtained from the hospital EMRs. The study was conducted over a period of 1 year.

### Sample size justification

**Figure 1:**
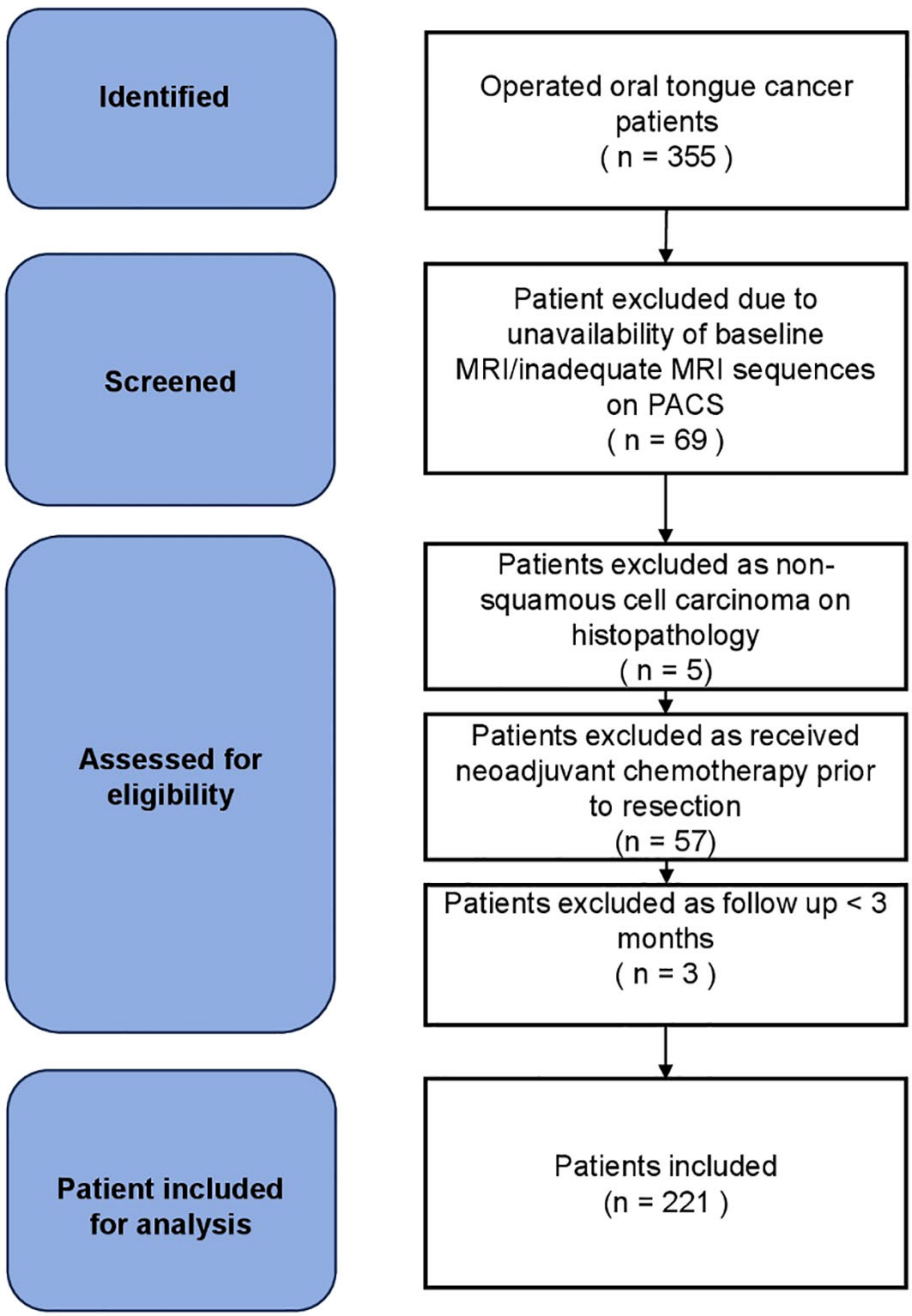
Flow diagram for selection of eligible patients.

A sample size calculation was not performed *a priori*; instead, a consecutive sampling approach was used to minimize selection bias. Between January 2021 and December 2022, 355 patients were screened, and 221 treatment-naive patients were included (**Figure 1**). This cohort was statistically adequate for survival analysis, as the 65 recorded events (deaths) for OS provided a sufficient events per variable (EPV) ratio. This adhered to the established “rule of ten”, ensuring the reliability of the hazard ratio estimates and preventing model overfitting in the multivariate Cox proportional hazards analysis.

### Inclusion criteria

Patients were included if a pre-treatment MRI had been performed within 4 weeks of upfront surgery for OTSCC. Inclusion also required the availability of good-quality, treatment-naive MR images in the picture archiving and communication system (PACS). Additionally, a post-operative histopathological report (HPR) needed to be available, containing details on pathological tumor (pT), PNI, and ENE.

### Exclusion criteria

Patients were excluded if essential MRI sequences were missing or if treatment-naive MRI images were not available. Additionally, cases were excluded if the tumor epicenter was located outside the oral tongue, if the follow-up period was less than 3 months, or if histopathological confirmation of recurrence was unavailable on the EMRs. **Figure 1** shows the flow diagram for the selection of eligible patients.

### Image analysis

Two blinded radiologists, having more than 10 and 5 years of experience in head and neck radiology, independently evaluated the baseline MRI images available on PACS for all 221 patients. Interobserver agreement between the two radiologists was assessed for the imaging biomarkers—peritumoral hyperenhancement, peritumoral edema, tumor ulceration, and extension up to <5 mm from the hyoid bone—and any discrepancies were resolved by mutual consensus. MRI images were acquired either in General Electric (GE) Signa Explorer 1.5T or Philips Ingenia 1.5T. All patients received 0.1 mmol/kg body weight of gadobenate dimeglumine using a contrast injector at 1.5–1.8 mL/s, followed by a saline flush at 2 mL/s. **Table 1** demonstrates the analyzed imaging parameters. The MRI protocol is outlined in **Supplementary Table 1**.

**Table 1:** Analyzed imaging parameters.

Feature and assessment details	Recorded as
Peritumoral hyperenhancement assessed on thin (≤2 mm) CE FSPGR sequences	Present/absent
Width of peritumoral hyperenhancement (if present) on thin (≤ 2 mm) axial CE FSPGR sequence	Measured in mm
Peritumoral edema evaluated on STIR as faint homogenous hyperintensity beyond heterogeneously hyperintense tumor margins	Present/absent
Tumor ulceration identified on any MRI sequence	Present/absent
Tumor extension up to <5 mm from the hyoid bone [shortest distance between tumor, including peritumoral hyperenhancement, and hyoid bone, measured on thin (≤ 2 mm) sagittal or coronal CE FSPGR by simultaneously evaluating the corresponding axial plane for tumor margins]	Present/absent

CE FSPGR, contrast-enhanced fast spoiled gradient echo; STIR, short tau inversion recovery; MRI, magnetic resonance imaging.

**Figure 2:**
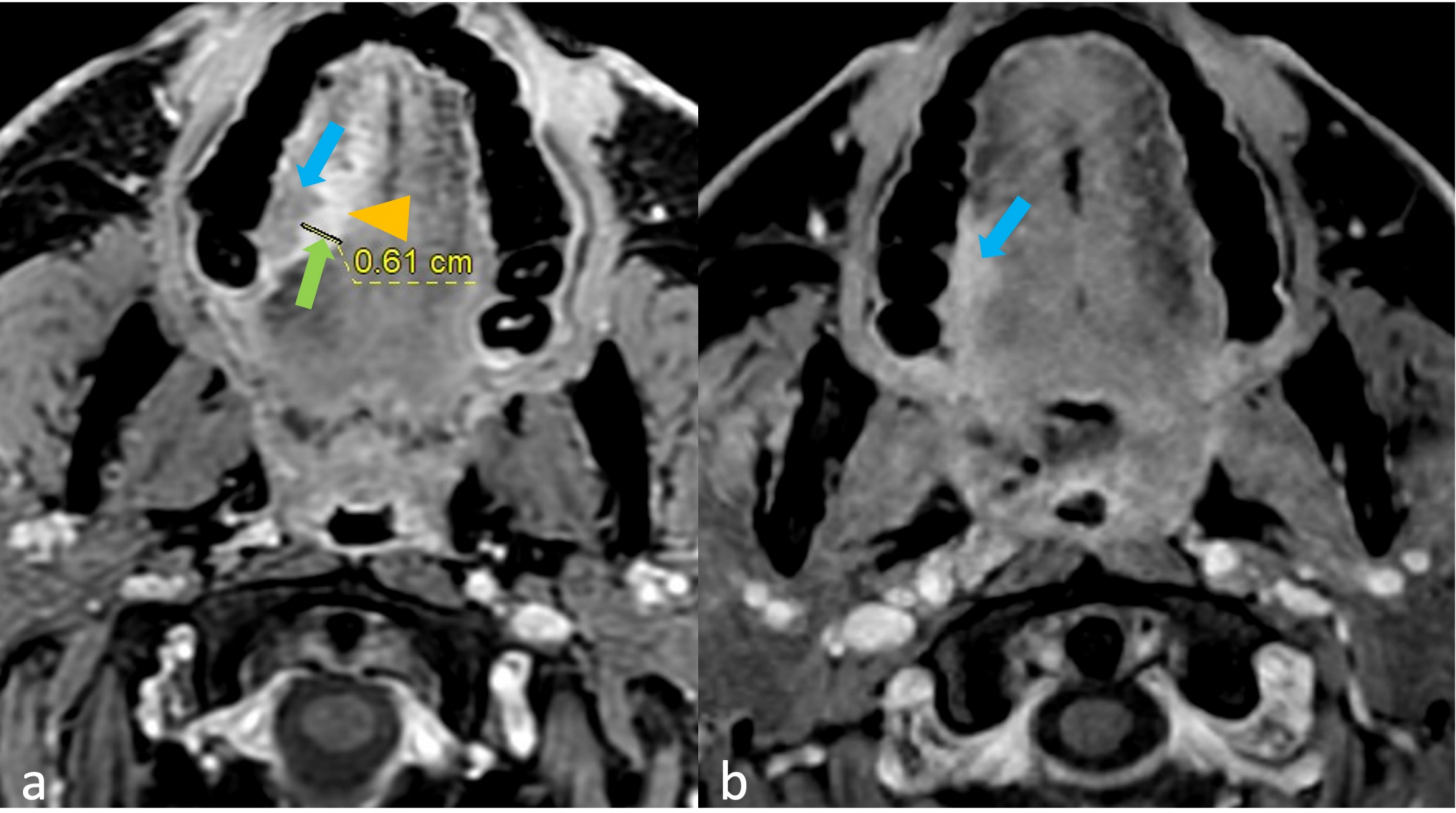
**(a, b)** Oral tongue squamous cell carcinomas with and without peritumoral hyperenhancement (PH) on MRI. **(a)** Axial thin contrast-enhanced (CE) fast spoiled gradient echo (FSPGR) sequence shows PH (arrowhead) surrounding the isoenhancing tumor (blue arrow). Measurement of maximum width of PH is 6.1 mm (green arrow), which is the PH width. **(b)** Axial thin CE FSPGR sequence shows an enhancing tumor without any PH (arrow).

**Figure 3:**
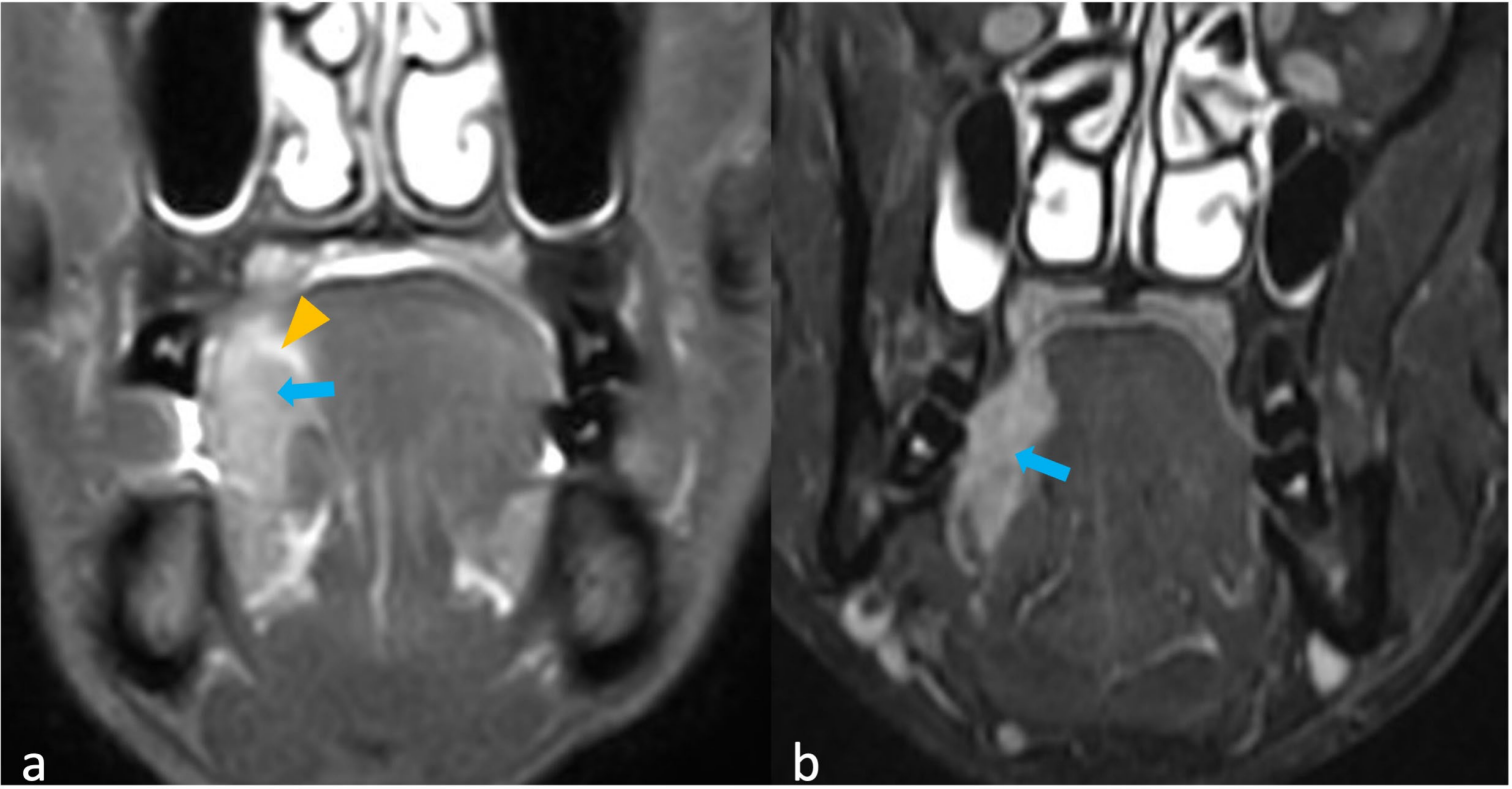
**(a, b)** MRI images of oral tongue squamous cell carcinomas with and without peritumoral edema (PE). **(a)** Coronal short tau inversion recovery (STIR) shows homogenously hyperintense PE (arrowhead) surrounding the heterogeneous right lateral border tumor (arrow). **(b)** Coronal STIR shows absence of PE surrounding the tumor (arrow).

**Figure 4:**
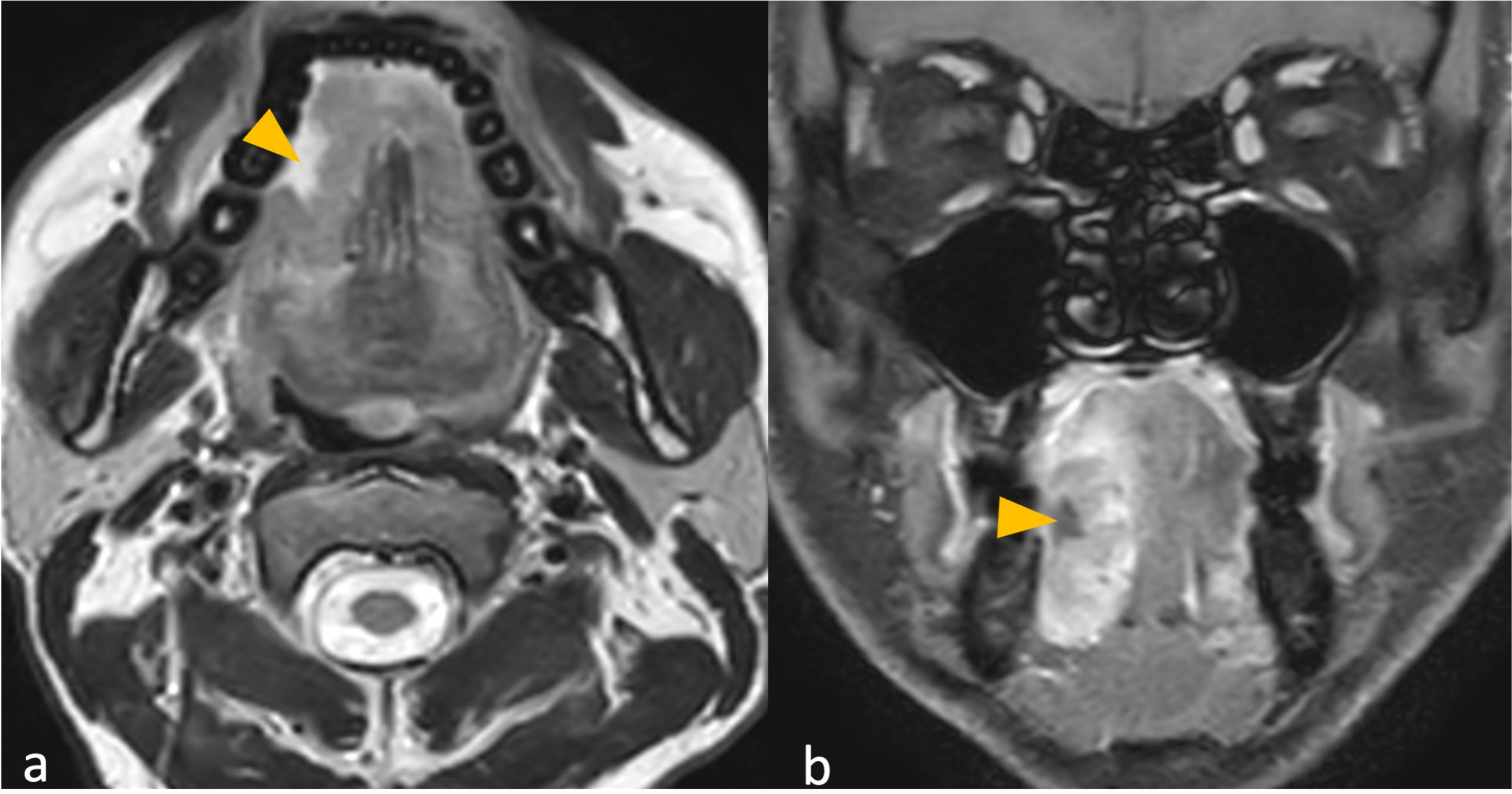
**(a, b)** MRI images of oral tongue squamous cell carcinoma with tumor ulceration. Axial T2 Weighted Imaging (T2WI) **(a)** and coronal contrast-enhanced (CE) fast spoiled gradient echo (FSPGR) image **(b)** show breach of mucosa overlying the tumor, suggestive of tumor ulceration (arrowheads).

**Figure 5:**
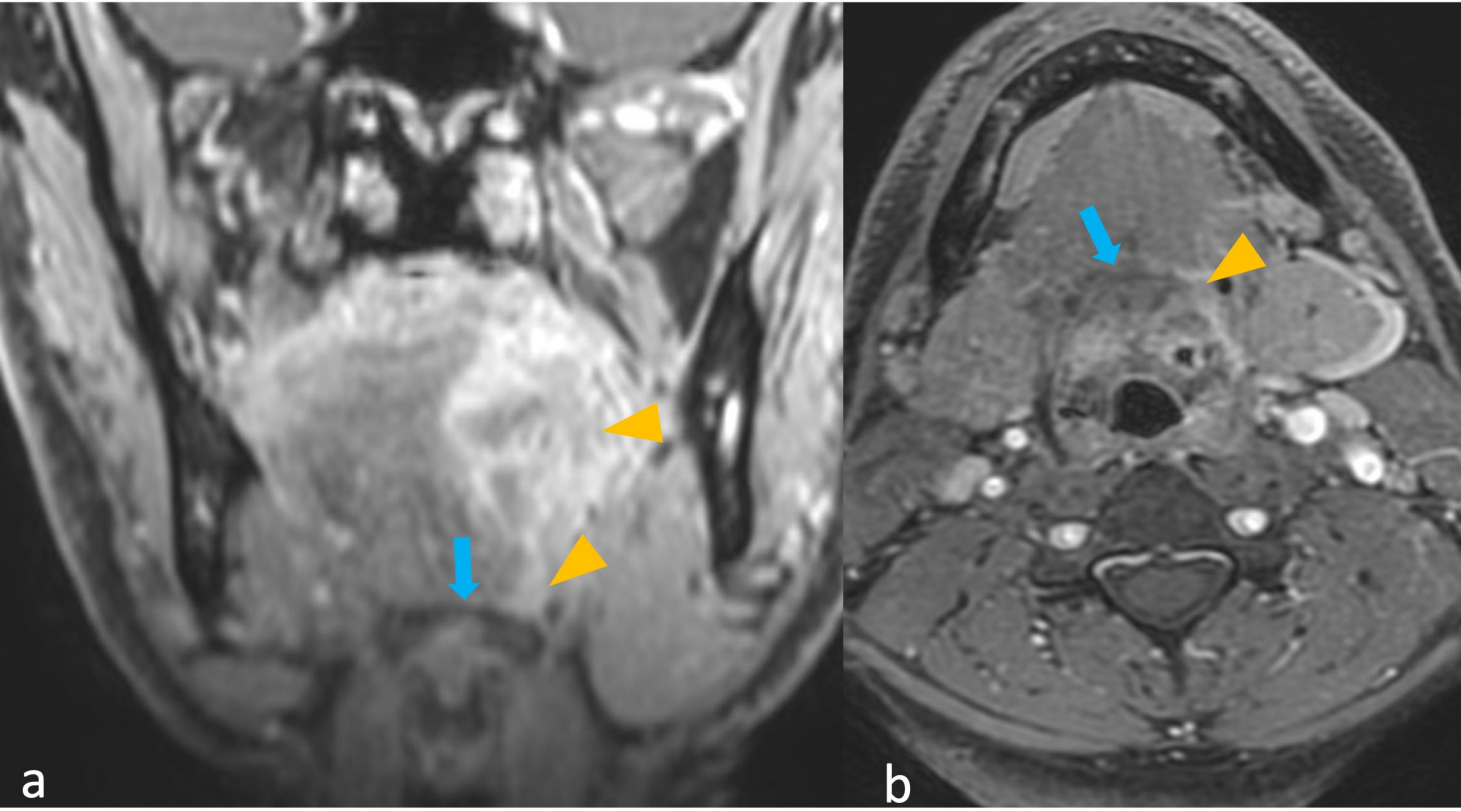
**(a, b)** MRI images of oral tongue squamous cell carcinoma with tumor reaching the hyoid bone. Coronal contrast-enhanced (CE) fast spoiled gradient echo (FSPGR) image **(a)** and axial CE FSPGR image **(b)** show tumor (arrowheads) extending to the hyoid bone (arrows).

**Figure 2** shows a tumor with and without peritumoral hyperenhancement and an example of the measurement of the maximum width of peritumoral hyperenhancement on MRI. **Figure 3** shows a tumor with and without peritumoral edema on MRI. **Figures 4**, **5** show the presence of tumor ulceration and extension of the tumor up to <5 mm from the hyoid bone, respectively.

### Post-operative pathological variables

Pathological variables such as pT category, PNI, and ENE were noted from the post-operative HPR available on EMRs.

### Follow-up

The 2-year follow-up data until 31/12/2024 were collected from EMRs for the calculation of OS, LRFS, LRRFS, DRFS, and DFS.

### Protocol for post-treatment follow-up and recurrence assessment

Patients at our institution were clinically followed up every 3 months after treatment completion for the first 2 years, followed by 6-monthly visits for the next 3 years. After completing 5 years, a follow-up was conducted annually. The choice of imaging modality—whether MRI (same protocol as baseline in **Supplementary Table 1**) or fluorodeoxyglucose positron emission tomography (FDG-PET) with contrast-enhanced computed tomography (CECT)—was guided by clinical suspicion and examination findings. A biopsy was performed as needed to confirm recurrence.

### Statistical methods

OS was defined as the time from the date of surgery to the date of death from any cause or last follow-up, expressed in months. DFS was defined as the time from the date of surgery to the date of first documented recurrence (local, regional, or distant) or death, whichever occurred first. LRFS was calculated from the date of surgery to the date of first local recurrence or last follow-up. Regional recurrence-free survival (RRFS) was calculated from the date of surgery to the date of first regional recurrence or last follow-up. Distant metastasis-free survival (DMFS) was defined as the time from the date of surgery to the date of first distant metastasis or last follow-up.

All time-to-event outcomes were calculated in months as the difference between the event date and the date of surgery. Patients without events were censored at the last follow-up date.

Descriptive analyses were conducted for demographic data such as sex, while continuous variables such as age were expressed as the mean ± standard deviation (SD) or as median [interquartile range (IQR)], depending on the normality of the data.

Survival outcomes were analyzed using the Kaplan–Meier method, and differences between groups were assessed using the log-rank test. The optimal cutoff value of peritumoral hyperenhancement width for predicting survival outcomes was determined using time-dependent receiver operating characteristic (ROC) analysis with the survival ROC package in R. Youden’s index was used to identify a cutoff value of peritumoral hyperenhancement width for predicting PNI and ENE. Univariate analyses of all imaging parameters were performed for survival outcomes, and parameters with significant *p*-values were included in multivariate Cox proportional hazards regression to estimate hazard ratios (HRs) with 95% confidence intervals (CIs).

Associations between categorical variables [e.g., tumor ulceration with PE, PH with pT categories, and PE with pT categories] were assessed using Fisher’s exact test or Pearson’s chi-squared test, as appropriate. Univariate binary logistic regression was performed to analyze the association between imaging parameters and PNI as well as ENE.

Interobserver agreement was assessed using Cohen’s kappa (K) statistic.

All statistical analyses were performed using SPSS version 25 (IBM, New York, USA) and R. A significance level of *p* < 0.05 was used.

## Results

### Demographic characteristics

The study comprised 179 men (81%) and 42 women (19%). The mean and median age of all the patients were 47.10 years ± 10.882 SD and 46 years (39–55 IQR), respectively.

### Tumor-related variables

A total of 179 patients had peritumoral hyperenhancement, 154 had peritumoral edema, 14 had tumor ulceration, and six patients had extension of tumor along the hyoglossus muscle up to <5 mm from the hyoid bone (three patients in the pT3 category, two in the pT2 category, and one in the pT4a category). There were no patients in the pT4b category.

Interobserver agreement between the two radiologists for peritumoral hyperenhancement, peritumoral edema, tumor ulceration, and extension of tumor up to <5 mm from the hyoid bone was 0.546 (*p* < 0.001), 0.628 (*p* < 0.001), 0.961 (*p* < 0.001), and 0.661 (*p* < 0.001), respectively, with tumor ulceration demonstrating almost perfect agreement.

### Survival analysis

The median follow-up was 32.62 months. A total of 63 patients had disease recurrence, of whom 11 had only local recurrence, nine had purely locoregional recurrence, five had local plus regional plus distant recurrence, one had local plus distant recurrence, 20 had purely regional recurrence, three had regional plus distant recurrence, and 14 had only distant recurrence.

**Figure 6:**
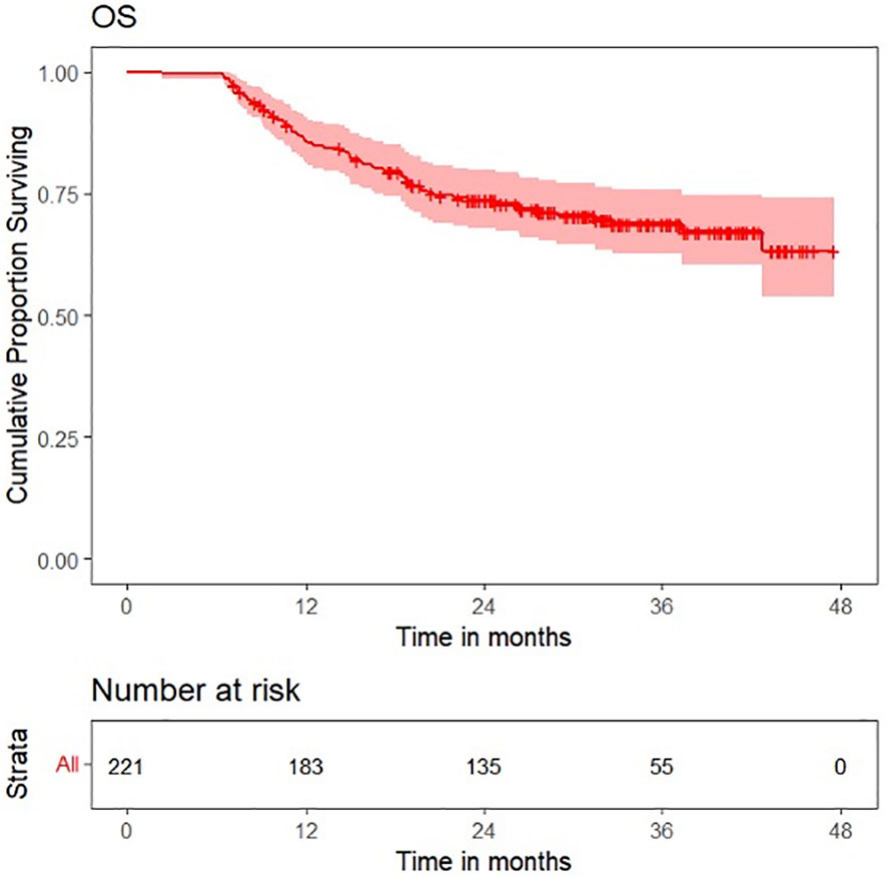
Survival plot for overall survival (OS).

**Figure 7:**
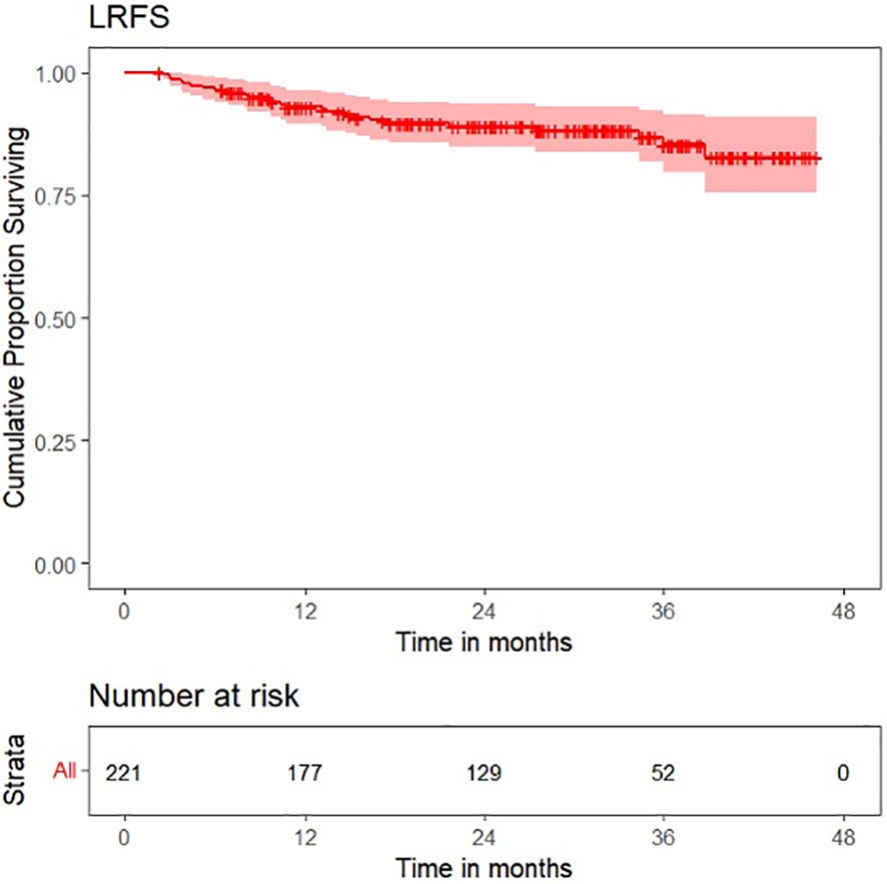
Survival plot for local recurrence-free survival (LRFS).

**Figure 8:**
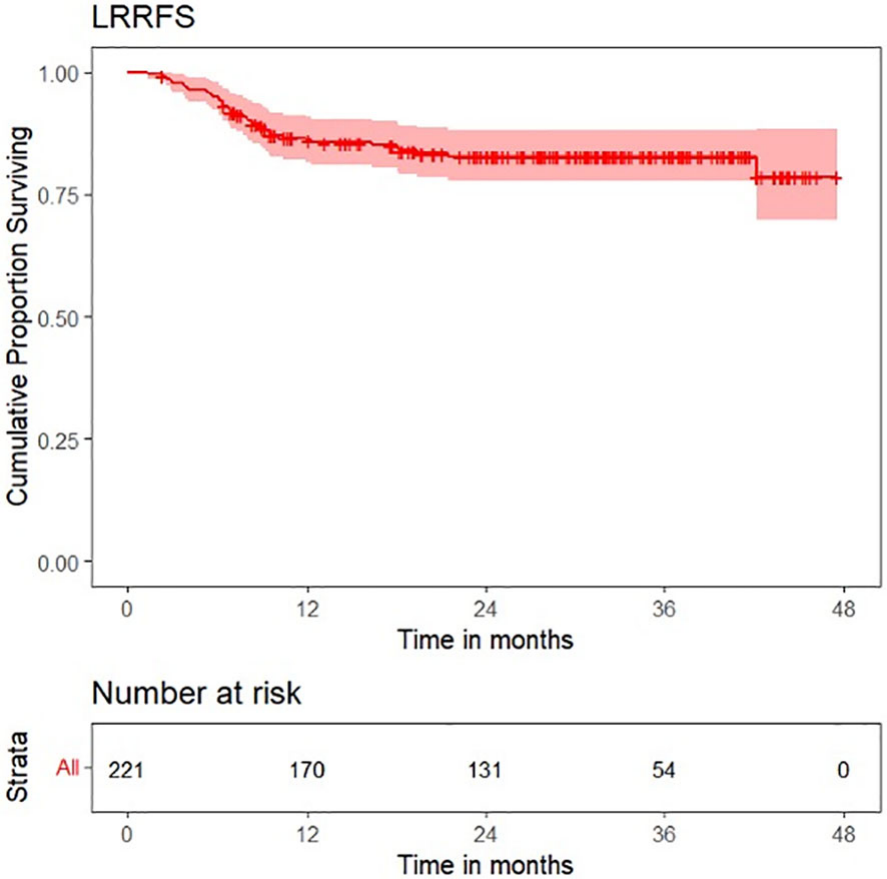
Survival plot for locoregional recurrence-free survival (LRRFS).

**Figure 9:**
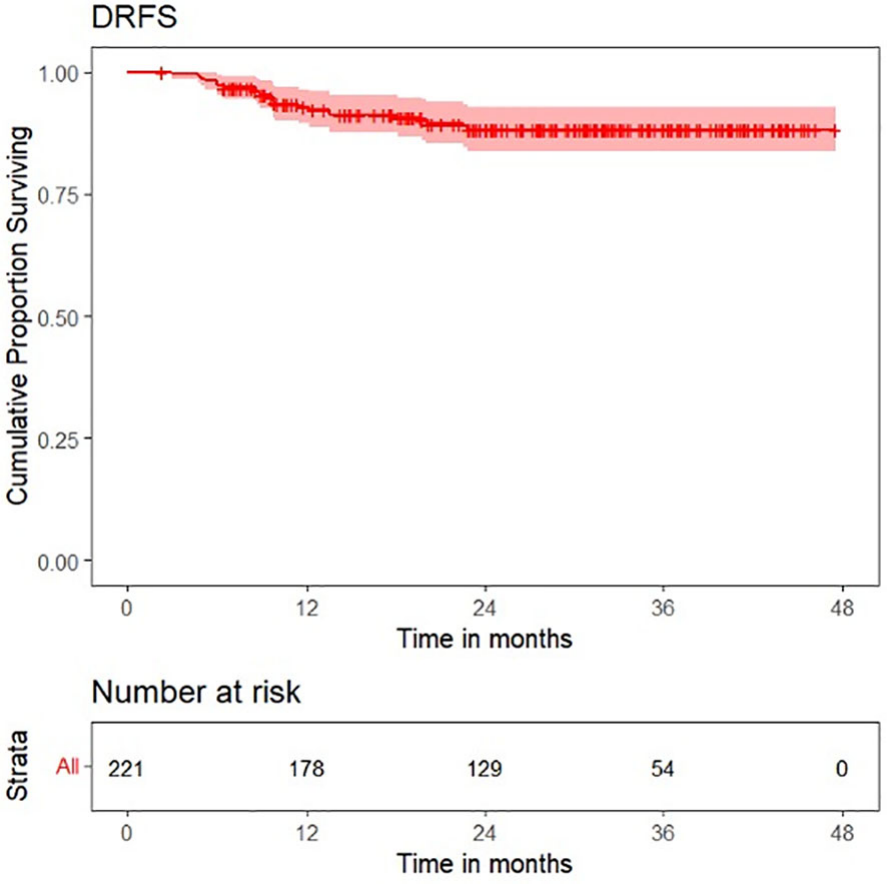
Survival plot for distant recurrence-free survival (DRFS).

**Figure 10:**
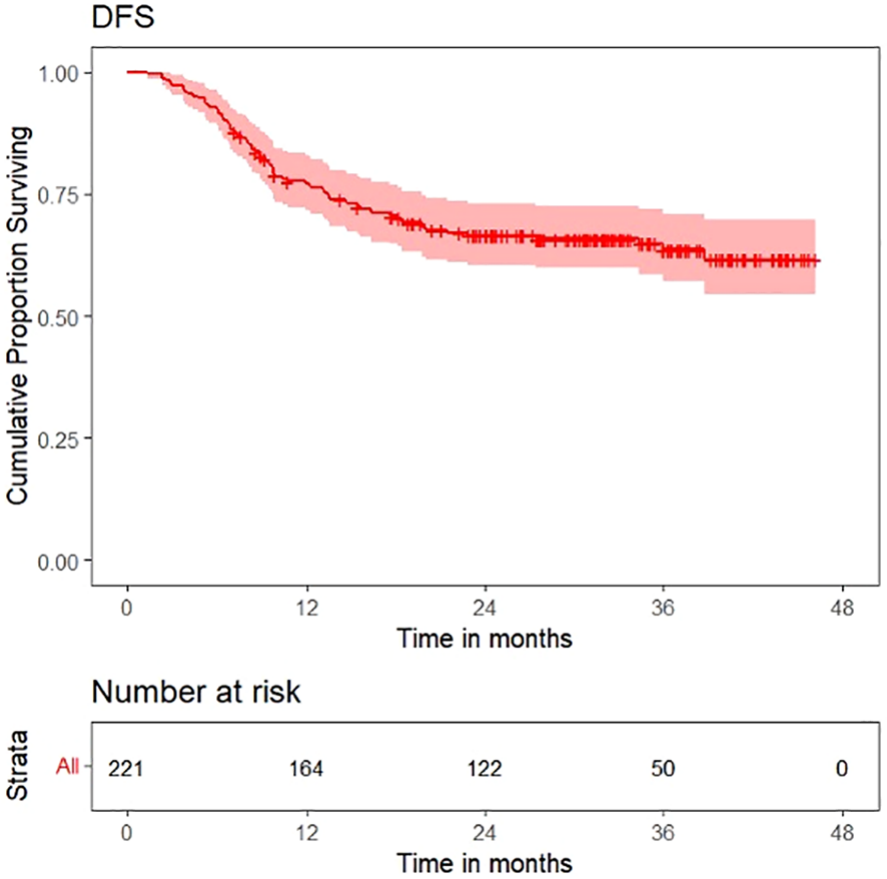
Survival plot for disease-free survival (DFS).

Survival plots for OS, LRFS, LRRFS, DRFS, and DFS are shown in **Figures 6**–**10**.

Worse OS was seen with peritumoral edema (HR: 2.43, 95% CI = 1.27 to 4.65; *p* = 0.007), peritumoral hyperenhancement (HR: 2.56, 95% CI = 1.10 to 5.92; *p* = 0.029), extension of tumor up to <5 mm from the hyoid bone (HR: 4.01, 95% CI = 1.45 to 11.11; *p* = 0.008), pathological T2 category (HR: 5.21, 95% CI = 1.59 to 17.14; *p* = 0.006), pathological T3 category (HR: 5.77, 95% CI = 1.73 to 19.23; *p* = 0.004), and pathological T4a category (HR: 12.07, 95% CI = 3.32 to 43.91; *p* < 0.001) with a significant *p*-value in univariate analysis (**Supplementary Table 2**).

Worse DRFS was seen with extension of tumor up to <5 mm from the hyoid bone (HR: 6.33, 95% CI = 1.46 to 27.5; *p* = 0.014), peritumoral edema (HR: 5.15, 95% CI = 1.21 to 22.0; *p* = 0.027), and pathological T4a category (HR: 6.54, 95% CI = 1.195 to 35.850; *p* = 0.030) with a significant *p*-value in univariate analysis (**Supplementary Table 3**).

Worse DFS was seen with extension of tumor up to <5 mm from the hyoid bone (HR: 4.58, 95% CI = 1.83 to 11.4; *p* = 0.001), tumor ulceration (HR: 2.21, 95% CI = 1.06 to 4.61; *p* = 0.034), peritumoral edema (HR: 2.00, 95% CI = 1.14 to 3.52; *p* = 0.016), pathological T2 category (HR: 4.52, 95% CI = 1.602 to 12.769; *p* = 0.004), pathological T3 category (HR: 5.11, 95% CI = 1.790 to 14.626; *p* = 0.002), and pathological T4a category (HR: 11.214, 95% CI = 3.610 to 34.841; *p* < 0.001) with a significant *p*-value in univariate analysis (**Supplementary Table 4**). None of the variables demonstrated a statistically significant *p*-value for LRFS or LRRFS in the univariate analysis.

**Figure 11:**
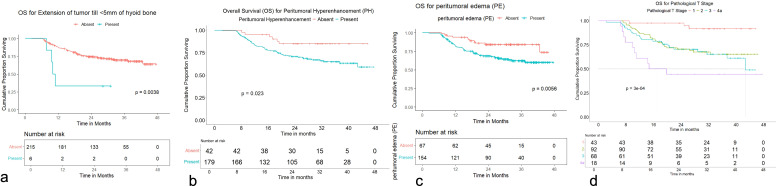
**(a–d)** Kaplan–Meier survival plots for overall survival (OS). **(a)** OS distribution for extension of tumor up to <5 mm from the hyoid bone. **(b)** OS distribution for peritumoral hyperenhancement. **(c)** OS distribution for peritumoral edema. **(d)** OS distribution for pathological T categories.

**Figure 12:**
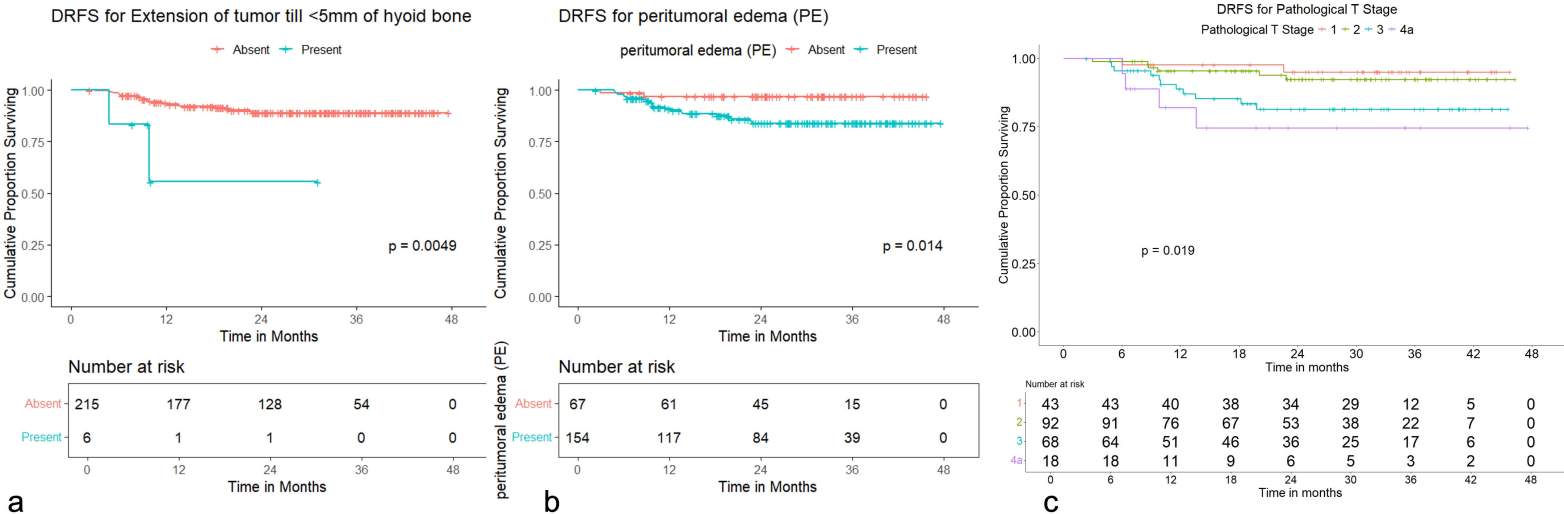
**(a–c)** Kaplan–Meier survival plots for distant recurrence-free survival (DRFS). **(a)** DRFS distribution for extension of tumor up to <5 mm from the hyoid bone. **(b)** DRFS distribution for peritumoral edema. **(c)** DRFS distribution for pathological T categories.

**Figure 13:**
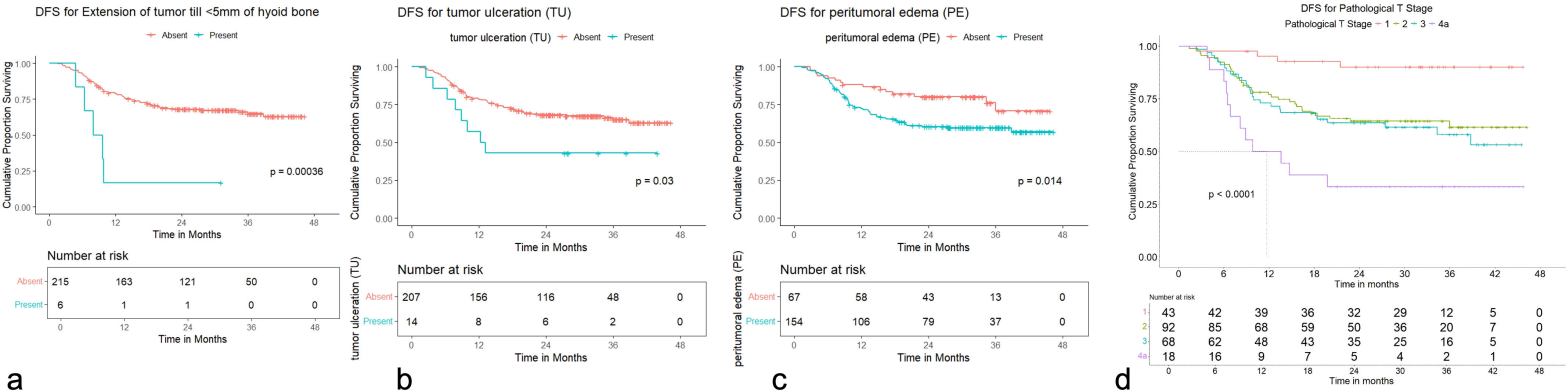
**(a–d)** Kaplan–Meier survival plots for disease-free survival (DFS). **(a)** DFS distribution for extension of tumor up to <5 mm from the hyoid bone. **(b)** DFS distribution for tumor ulceration. **(c)** DFS distribution for peritumoral edema. **(d)** DFS distribution for pathological T categories.

OS distribution for peritumoral edema, peritumoral hyperenhancement, extension of tumor up to <5 mm from the hyoid bone, and pathological T categories on the Kaplan–Meier survival plots are shown in **Figure 11**. DRFS distribution for extension of tumor up to <5 mm from the hyoid bone, peritumoral edema, and pathological T categories on the Kaplan–Meier survival plots are shown in **Figure 12**. DFS distribution for extension of tumor up to <5 mm from the hyoid bone, tumor ulceration, peritumoral edema, and pathological T categories on the Kaplan–Meier survival plots are shown in **Figure 13**.

In multivariate analysis, worse OS with a significant *p*-value was seen for extension of tumor up to <5 mm from the hyoid bone (HR: 3.05, 95% CI = 1.09 to 8.54; *p* = 0.034), pathological T2 category (HR: 6.98, 95% CI = 1.48 to 32.80; *p* = 0.014), pathological T3 category (HR: 10.39, 95% CI = 1.81 to 59.47; *p* = 0.009), and pathological T4a category (HR: 21.6, 95% CI = 3.34 to 139.51; *p* = 0.001). Worse DRFS with a significant *p*-value was seen for extension of tumor up to <5 mm from the hyoid bone (HR: 4.66, 95% CI = 1.07 to 20.32; *p* = 0.041) and the presence of peritumoral edema (HR: 4.77, 95% CI = 1.11 to 20.50; *p* = 0.036). Worse DFS with a significant *p*-value was seen for extension of tumor up to <5 mm from the hyoid bone (HR: 3.68, 95% CI = 1.43 to 9.42; *p* = 0.007), pathological T2 category (HR: 5.66, 95% CI = 1.40 to 22.97; *p* = 0.015), pathological T3 category (HR: 8.71, 95% CI = 1.78 to 42.66; *p* = 0.008), and pathological T4a category (HR: 17.94, 95% CI = 3.28 to 98.19; *p* = 0.001).

### Association between variables

All tumor ulcerations had peritumoral edema. All pT4a patients showed peritumoral hyperenhancement, whereas peritumoral edema was absent in only one patient with pT4a. The pathological primary tumor category-wise distribution of peritumoral hyperenhancement and peritumoral edema is shown in **Supplementary Tables 5**, **6**, respectively.

Significant association was seen between peritumoral hyperenhancement [odds ratio 95% CI = 9.11 (2.13–39.01), *p* = 0.003], peritumoral edema [odds ratio 95% CI = 3.55 (1.57–7.98), *p* = 0.002], tumor ulceration [odds ratio 95% CI = 3.06 (1.02–9.14), *p* = 0.045], extension of tumor up to <5 mm from the hyoid bone [odds ratio 95% CI = 5.96 (1.06–33.47, *p* = 0.042], and ENE (**Table 2**). None of these variables showed a significant association with PNI.

**Table 2:** Association between categorical variables on imaging and extranodal extension (ENE).

Variable	Extranodal extension (ENE)			Odds ratio (95% CI)	*p*-Value
	Overall, N = 221	Absent, N = 163	Present, N = 59		
Extension of tumor up to <5 mm from the hyoid bone					
Absent	215 (97.3)	161 (98.8)	54 (93.1)	1 (Reference)	**0.042**
Present	6 (2.7)	2 (1.2)	4 (6.9)	5.96 (1.06–33.47)	
Tumor ulceration					
Absent	207 (93.7)	156 (95.7)	51 (87.9)	1 (Reference)	**0.045**
Present	14 (6.3)	7 (4.3)	7 (12.1)	3.06 (1.02–9.14)	
Peritumoral hyperenhancement					
Absent	42 (19.0)	40 (24.5)	2 (3.4)	1 (Reference)	**0.003**
Present	179 (81.0)	123 (75.5)	56 (96.6)	9.11 (2.13–39.01)	
Peritumoral hyperenhancement width					
≤1.25 mm	13 (7.3)	11 (8.9)	2 (3.6)	1 (Reference)	0.206
>1.25 mm	166 (92.7)	112 (91.1)	54 (96.4)	2.70 (0.58–12.61)	
Peritumoral edema					
Absent	67 (30.3)	59 (36.2)	8 (13.8)	1 (Reference)	**0.002**
Present	154 (69.7)	104 (63.8)	50 (86.2)	3.55 (1.57–7.98)	

Significant P values have been highlighted in bold.

Cutoff values of 2.65 mm and 1.25 mm were identified for peritumoral hyperenhancement width for predicting PNI and ENE, respectively. Patients with peritumoral hyperenhancement width >2.65 mm had a significantly higher frequency of PNI compared to those with ≤2.65 mm (48.1% vs. 25.5%, *p* = 0.001). There was a significant association between the peritumoral hyperenhancement threshold of 2.65 mm and pT categories (*p* < 0.001). Patients with width >1.25 mm showed a higher prevalence of ENE (96.4% vs. 91.1%), although this difference was not statistically significant (**Table 2**).

As the evaluated imaging biomarkers—peritumoral hyperenhancement, peritumoral edema, tumor ulceration, and extension up to <5 mm from the hyoid bone—do not have direct histopathological correlates, their diagnostic performance was assessed for predicting pathological ENE, a well-established adverse prognostic factor. This was performed by calculating sensitivity, specificity, positive predictive value (PPV), negative predictive value (NPV), and overall accuracy (**Table 3**).

**Table 3:** Diagnostic performance of imaging biomarkers for predicting pathological extranodal extension.

Imaging biomarker	Sensitivity	Specificity	Accuracy	PPV	NPV
Peritumoral hyperenhancement	96.60%	24.50%	43.40%	31.30%	95.20%
Peritumoral edema	86.20%	36.20%	49.30%	32.50%	88.10%
Tumor ulceration	12.10%	95.70%	73.80%	50.00%	75.40%
Extension up to <5 mm from the hyoid bone	6.90%	98.80%	75.10%	66.70%	74.90%

PPV, positive predictive value; NPV, negative predictive value.

## Discussion

Peritumoral edema is associated with significantly worse DRFS in multivariate analysis. Although tumor ulceration shows a significant association with worse DFS only in univariate analysis, all patients with tumor ulceration also had peritumoral edema, underscoring its adverse prognostic impact. Extension of the tumor up to <5 mm from the hyoid bone is associated with significantly worse OS, DRFS, and DFS in multivariate analysis. Although only six patients demonstrated tumor extension up to <5 mm from the hyoid bone, they were distributed across pathological T2, T3, and T4a categories, suggesting that this finding may represent an independent marker of poor prognosis. These variables, along with peritumoral hyperenhancement, are significantly linked to ENE. Peritumoral hyperenhancement width >2.65 mm is significantly associated with PNI.

Jani et al. (12) looked into the presence or absence of peritumoral enhancement along with patterns of enhancement on MRI of 77 patients with tongue carcinoma, and they found worse DFS with the presence of peritumoral enhancement on univariate analysis, while we found worse OS in the univariate analysis in those with peritumoral hyperenhancement. Peritumoral edema on pre-treatment MRI has been associated with poor prognosis in breast cancer, glioblastoma multiforme, and hepatocellular carcinoma; however, its prognostic significance in head and neck cancers, including OTSCC, remains unexplored (13–16). Tumors extending up to the hyoid bone are generally considered technically unresectable; however, no formal MRI study has yet evaluated their prognostic significance (17, 18). The prognostic significance of tumor ulceration in oral tongue cancer has not yet been evaluated.

Studies have shown that pathological DOI is a predictor of PNI and ENE; however, to the best of our knowledge, the association between peritumoral edema, peritumoral hyperenhancement width, tumor ulceration, extension of tumor up to <5 mm from the hyoid bone, and ENE and PNI has not been explored (19, 20).

Our findings suggest that pre-operative MRI can provide valuable prognostic information beyond conventional staging, allowing for early identification of patients at higher risk for recurrence and poor survival. The integration of these imaging biomarkers into routine pre-operative assessment may facilitate more personalized treatment planning, including consideration of intensified adjuvant therapy or closer follow-up for high-risk patients. Furthermore, the correlation of peritumoral hyperenhancement width >2.65 mm with pathological features such as PNI, and the correlation between peritumoral hyperenhancement, peritumoral edema, tumor ulceration, and pathological features such as ENE, underscore the potential of imaging to reflect underlying tumor biology and aggressiveness, highlighting the role of radiomics and quantitative imaging in future research.

The strength of our study lies in the fact that a large, homogenous oral tongue carcinoma cohort treated with surgery was evaluated over 2 years at a single tertiary cancer center, and several novel imaging parameters were assessed for the first time in relation to survival outcomes. The prognosis of OTSCC is generally poor, and it is difficult to prognosticate locally advanced OTSCC. These new imaging parameters may hold promise for guiding treatment intensification in patients with worse prognostic features in future prospective studies.

One of the limitations of this study was the small number of cases demonstrating tumor extension within 5 mm from the hyoid bone, observed in only six patients. Therefore, validation through prospective studies with larger cohorts is necessary. Additionally, external validation across multiple centers would be valuable to confirm the reproducibility and generalizability of these imaging biomarkers. Surgical outcomes are influenced by the technique and expertise of the procedure, while host factors such as immunity, nutrition, and habits can affect the recurrence of tongue carcinoma; however, these variables were not considered in this study.

## Conclusion

Peritumoral edema, tumor ulceration, peritumoral hyperenhancement, peritumoral hyperenhancement width >2.65 mm, and tumor extension to <5 mm from the hyoid bone are adverse imaging biomarkers for oral tongue squamous cell carcinoma. These parameters hold significant potential to refine risk stratification and could inform future T4 staging, warranting prospective validation in larger cohorts.

## Data Availability

The original contributions presented in the study are included in the article/**Supplementary Material**. Further inquiries can be directed to the corresponding author.
