# Supplementary material for: MRI-based tumor signatures as prognostic biomarkers in oral tongue squamous cell carcinoma
Source: Front Oncol. 2026 Apr 14;16:1809223. doi: 10.3389/fonc.2026.1809223 (PMC13121357; doi:10.3389/fonc.2026.1809223)
Supplement: Supplementary file 1 [file Table1.docx]

**Supplementary Tables**

**Supplementary Table 1: MRI protocol parameters**

| **Sequences and plane** | **Slice thickness** | **Spacing** | **Field of view** |
| --- | --- | --- | --- |
| Coronal STIR | 4 mm | 1 mm | 230 mm |
| Coronal T1WI | 4 mm | 1 mm | 230 mm |
| Sagittal T2WI | 4 mm | 1 mm | 240 mm |
| Axial T1WI | 3 mm | 0.4 mm | 160 mm |
| Axial T2WI | 3 mm | 0.4 mm | 160 mm |
| Axial T2WI | 4 mm | 1 mm | 220 mm |
| DWI (b-values 0 and 800 s/mm^2^) | 4 mm | 1 mm | 300 mm |
| Axial T1 post contrast fat saturated | 3 mm | 0.4 mm | 160 mm |
| Coronal T1 post contrast fat saturated | 4 mm | 1 mm | 230 mm |
| Axial post contrast 3D FSPGR | < 2 mm | 0 | 230 mm |
| STIR- Short tau inversion recovery, WI- Weighted Image, DWI- Diffusion Weighted Image, 3D FSPGR-Three Dimensional Fast Spoiled Gradient Echo (FSPGR) sequences | | | |

**Supplementary Table 2: Univariate analysis for Overall Survival**

| **Characteristic** | **N** | **Event N** | **Hazard Ratio** | **95% Confidence Interval** | **p-value** |
| --- | --- | --- | --- | --- | --- |
| **Extension of tumor till < 5 mm of hyoid bone** |  |  |  |  |  |
| Absent | 215 | 61 | Reference |  |  |
| Present | 6 | 4 | 4.01 | 1.45, 11.11 | **0.008** |
| **Tumor ulcer** |  |  |  |  |  |
| Absent | 207 | 59 | Reference |  |  |
| Present | 14 | 6 | 1.46 | 0.63, 3.40 | 0.373 |
| **Peritumoral hyperenhancement** |  |  |  |  |  |
| Absent | 42 | 6 | Reference |  |  |
| Present | 179 | 59 | 2.56 | 1.10, 5.92 | **0.029** |
| **Peritumoral hyperenhancement width** |  |  |  |  |  |
| < 2.35 | 96 | 30 | Reference |  |  |
| >2.35 | 83 | 29 | 1.19 | 0.72, 2.00 | 0.489 |
| **Peritumoral edema** |  |  |  |  |  |
| Absent | 67 | 11 | Reference |  |  |
| Present | 154 | 54 | 2.43 | 1.27, 4.65 | **0.007** |
| **Pathological T stage** |  |  |  |  |  |
| T1 | 43 | 3 | Reference |  |  |
| T2 | 92 | 29 | 5.21 | 1.59, 17.14 | **0.006** |
| T3 | 68 | 23 | 5.77 | 1.73, 19.23 | **0.004** |
| T4a | 18 | 10 | 12.07 | 3.32, 43.91 | **<0.001** |

**Supplementary Table 3: Univariate analysis for Distant Recurrence Free Survival**

| **Characteristic** | **N** | **Event N** | **Hazard Ratio** | **95% Confidence Interval** | **p-value** |
| --- | --- | --- | --- | --- | --- |
| **Extension of tumor till < 5 mm of hyoid bone** |  |  |  |  |  |
| Absent | 215 | 21 | Reference |  |  |
| Present | 6 | 2 | 6.33 | 1.46, 27.5 | **0.014** |
| **Tumor ulceration** |  |  |  |  |  |
| Absent | 207 | 20 | Reference |  |  |
| Present | 14 | 3 | 2.20 | 0.65, 7.41 | 0.203 |
| **Peritumoral hyperenhancement** |  |  |  |  |  |
| Absent | 42 | 3 | Reference |  |  |
| Present | 179 | 20 | 1.74 | 0.52, 5.85 | 0.372 |
| **Peritumoral hyperenhancement width** |  |  |  |  |  |
| < 2.35 | 96 | 12 | Reference |  |  |
| >2.35 | 83 | 8 | 0.79 | 0.32, 1.92 | 0.596 |
| **Peritumoral edema** |  |  |  |  |  |
| Absent | 67 | 2 | Reference |  |  |
| Present | 154 | 21 | 5.15 | 1.21, 22.0 | **0.027** |
| **Pathological T stage** |  |  |  |  |  |
| T1 | 43 | 2 | Reference |  |  |
| T2 | 92 | 6 | 1.55 | 0.313, 7.696 | 0.590 |
| T3 | 68 | 11 | 4.17 | 0.924, 18.841 | 0.063 |
| T4a | 18 | 4 | 6.54 | 1.195, 35.850 | **0.030** |

**Supplementary Table 4: Univariate analysis for Disease Free Survival**

| **Characteristic** | **N** | **Event N** | **Hazard Ratio** | **95% Confidence Interval** | **p-value** |
| --- | --- | --- | --- | --- | --- |
| **Extension of tumor till < 5 mm of hyoid bone** |  |  |  |  |  |
| Absent | 215 | 71 | Reference |  |  |
| Present | 6 | 5 | 4.58 | 1.83, 11.4 | **0.001** |
| **Tumor ulceration** |  |  |  |  |  |
| Absent | 207 | 68 | Reference |  |  |
| Present | 14 | 8 | 2.21 | 1.06, 4.61 | **0.034** |
| **Peritumoral hyperenhancement** |  |  |  |  |  |
| Absent | 42 | 10 | Reference |  |  |
| Present | 179 | 66 | 1.73 | 0.89, 3.36 | 0.108 |
| **Peritumoral hyperenhancement width** |  |  |  |  |  |
| < 2.35 | 96 | 33 | Reference |  |  |
| >2.35 | 83 | 33 | 1.22 | 0.75, 1.97 | 0.426 |
| **Peritumoral edema** |  |  |  |  |  |
| Absent | 67 | 15 | Reference |  |  |
| Present | 154 | 61 | 2.00 | 1.14, 3.52 | **0.016** |
| **Pathological T stage** |  |  |  |  |  |
| T1 | 43 | 4 | Reference |  |  |
| T2 | 92 | 33 | 4.52 | 1.602, 12.769 | **0.004** |
| T3 | 68 | 27 | 5.11 | 1.790, 14.626 | **0.002** |
| T4a | 18 | 12 | 11.214 | 3.610, 34.841 | **<0.001** |

**Supplementary Table 5: Pathological primary tumor category-wise distribution of Peritumoral hyperenhancement**

| **Label** | **Variable** | **Pathological T categories 1/2/3/4a/4b** | | | | **Total** | **Test** |
| --- | --- | --- | --- | --- | --- | --- | --- |
|  |  | **1** | **2** | **3** | **4a** |  |  |
| **PH** | Absent | 18 (42.85%) | 19 (45.23%) | 5 (11.90%) | 0 (0%) | 42 (19%) | p value: <0.000  (Fisher's Exact Test for Count Data) |
|  | Present | 25 (13.96%) | 73 (40.78%) | 63 (35.19%) | 18 (10.05%) | 179 (80.99%) |  |
|  | Total | 43 (19.45%) | 92 (41.62%) | 68 (30.76%) | 18 (8.14%) | 221 (100.00%) |  |
| PH: Peritumoral hyperenhancement | | | | | | | |

**Supplementary Table 6: Pathological primary tumor category-wise distribution of Peritumoral edema**

| **Label** | **Variable** | **Pathological T categories 1/2/3/4a/4b** | | | | **Total** | **Test** |
| --- | --- | --- | --- | --- | --- | --- | --- |
|  |  | **1** | **2** | **3** | **4a** |  |  |
| **PE** | Absent | 26 (38.80%) | 30 (44.77%) | 10 (14.92%) | 1 (1.49%) | 67 (30.31%) | p value: <0.000 (Pearson's Chi-squared test) |
|  | Present | 17 (11.03%) | 62 (40.25%) | 58 (37.66%) | 17 (11.03%) | 154 (69.68%) |  |
|  | Total | 43 (19.45%) | 92 (41.62%) | 68 (30.76%) | 18 (8.14%) | 221 (100.00%) |  |
| PE: Peritumoral edema | | | | | | | |
